# Integrin Based Isolation Enables Purification of Murine Lineage Committed Cardiomyocytes

**DOI:** 10.1371/journal.pone.0135880

**Published:** 2015-08-31

**Authors:** Laura Tarnawski, Xiaojie Xian, Gustavo Monnerat, Iain C. Macaulay, Daniela Malan, Andrew Borgman, Sean M. Wu, Bernd K. Fleischmann, Stefan Jovinge

**Affiliations:** 1 Lund Strategic Research Center for Stem Cell Biology and Cell Therapy, Lund University, Lund, Sweden; 2 Van Andel Research Institute, Grand Rapids, Michigan, United States of America; 3 Institute of Physiology I, Life and Brain Center, Department of Cardiac Surgery, University of Bonn, Bonn, Germany; 4 Institute of Biophysics Carlos Chagas Filho, Federal University of Rio de Janeiro, Rio de Janeiro, Brazil; 5 Haematopoietic Stem Cell Laboratory, Weatherall Institute of Molecular Medicine, University of Oxford, Oxford, England; 6 Institute for Stem Cell Biology and Regenerative Medicine, Stanford, California, United States of America; 7 Stanford Cardiovascular Institute, Stanford, California, United States of America; 8 Dept of Medicine, Division of Cardiology, Stanford University School of Medicine, Stanford, California, United States of America; 9 Pharma Center Bonn, University of Bonn, Bonn, Germany; 10 Spectrum Health Fredrik Meijer Heart and Vascular Institute, Grand Rapids, Michigan, United States of America; University of Milan, ITALY

## Abstract

In contrast to mature cardiomyocytes which have limited regenerative capacity, pluripotent stem cells represent a promising source for the generation of new cardiomyocytes. The tendency of pluripotent stem cells to form teratomas and the heterogeneity from various differentiation stages and cardiomyocyte cell sub-types, however, are major obstacles to overcome before this type of therapy could be applied in a clinical setting. Thus, the identification of extracellular markers for specific cardiomyocyte progenitors and mature subpopulations is of particular importance. The delineation of cardiomyocyte surface marker patterns not only serves as a means to derive homogeneous cell populations by FACS, but is also an essential tool to understand cardiac development. By using single-cell expression profiling in early mouse embryonic hearts, we found that a combination of integrin alpha-1, alpha-5, alpha-6 and N-cadherin enables isolation of lineage committed murine cardiomyocytes. Additionally, we were able to separate trabecular cardiomyocytes from solid ventricular myocardium and atrial murine cells. These cells exhibit expected subtype specific phenotype confirmed by electrophysiological analysis. We show that integrin expression can be used for the isolation of living, functional and lineage-specific murine cardiomyocytes.

## Introduction

Cell transplantation therapy to treat heart disease has become more promising due to the recent developments in pluripotent stem cell derived cardiomyocytes. However, transplantation of heterogeneous stem cell derived cell populations carries with it the risk of teratoma formation. Furthermore, due to the heart’s electrophysiological heterogeneity highly purified progenitor populations are needed in order to minimize the risk of arrhythmias. We have previously established a sorting strategy for fetal cardiomyocytes based on characterization of their surface-markers [1] and thereby proving the concept that viable cardiomyocytes can be isolated by fluorescence-activated cell sorting (FACS).

While this was the first step in isolating a pure pan-cardiomyocyte population, additional markers are necessary for the segregation of atrial and ventricular sub-populations. Thus, the primary purpose of this study was to identify surface markers for isolating pure embryonic atrial and ventricular subpopulations maintaining their subtype specific physiology.

Two markers that initially specify both endothelial and cardiac cells are the kinase insert domain protein receptor (FLK1) and the calcium-dependent cell adhesion molecule (CDH2). Early during cardiac development Flk1 expression becomes confined to endothelial cells. Cdh2 expression, on the other hand, decreases in endocardial precursors [2] but continues to be expressed in all cardiomyocytes throughout development [3]. Cdh2 is thus a more versatile cardiac marker than VCAM-1, a cardiomyocyte-specific marker during development, which loses its cardiac specificity after embryonic day 13.5 [1]. As Cdh2 is expressed in all cardiomyocytes, other markers are necessary for the segregation of atrial and ventricular sub-populations. Myosin light chain 2v (Myl2) is one of the most studied and well-defined intracellular cardiomyocyte markers and it is expressed in most of the heart tube during development. Its expression gradually becomes restricted to the primitive ventricles and outflow tract [4]. Myosin light chain 2A (Myl7) on the other hand, is initially expressed throughout the myocardium and becomes restricted to the atrial myocardium as late as ED12.5, with expression fading in the left ventricle earlier than the right [5, 6]. As intracellular markers, however, Myl2 and Myl7 cannot be used for live cell sorting. In contrast to myosin chamber specificity, the members of the integrin receptor family are to some degree expressed on all nucleated mammalian cells [7] as they are the cells’ main communicators to the extracellular matrix [8] and to adjoining cells [7]. Cardiomyocytes express a wide variety of integrins, including integrin alpha-1 (Itga1), alpha-5 (Itga5) and alpha-6 (Itga6) [8, 9]. It has previously been shown that Itga6 is strongly expressed in the atrial and trabecular ventricular myocardium but absent from the compact layer of the ventricles [10, 11]. Both Itga1 and Itga5 have been detected in fetal and neonatal murine cardiomyocytes, but have been reported to be absent in adult hearts [12]. Even though their temporal and spatial expression has been studied, it remains unclear to what extent integrins can be exploited to discriminate cardiomyocyte subpopulations.

In this paper we delineate the Itga6, Itga1 and Itga5 expression pattern of murine cardiomyocytes, and more importantly, establish a FACS based protocol for their isolation with high viability and high purity. Since the marker delineation was made during early development, these markers are of special interest for the use in deriving cells from pluripotent cell sources. Moreover, cardiomyocytes during early development are especially relevant for transplantation purposes since they are more resistant to ischemia and more readily divide compared to adult cardiomyocytes [13]. This would consequently enable isolation of cells from induced pluripotent stem cell sources that will be vital for future applications to the cell transplantation field.

## Methods

### Animals and ethics statement

This study was approved by and all animals were housed and treated in accordance with the Local Swedish Animal Ethics Committee at Lund University (permit number M270-11), the Directive 2010/63/EU of the European Parliament and the National Institutes of Health (NIH) Guide for the Care and Use of Experimental Animals and approvals from the Institutional Animal Care and Use Committee (IACUC, protocol number: 14-08-024) of the Van Andel Research Institute. Wild-type C57/BL6JBomBmsd (Charles River) or heterozygous embryos derived from crossings between homozygous Nkx2.5-eGFP cardiac enhancer transgenic males and C57/BL6JBomBmsd females were used. The Nkx2.5-eGFP strain was backcrossed onto a C57/BL6J background. The mice were housed in facilities running 12/12 light/dark cycles at temperatures of 22–23°C and had continuous access to water and food. These conditions were maintained throughout the duration of the study and all efforts were made to minimize suffering. Euthanasia was performed by carbon dioxide followed by immediate cervical dislocation.

### Tissue dissociation

Hearts from mouse embryos were dissociated into single cell suspension in isolation buffer containing (mmol/L); 130 NaCl, 5 KCL, 1.2 KH_2_PO_4_, 6 HEPES, 5 NaHCO_3_, 1 MgSO_4_ and 5 Glucose (pH 7.5) supplemented with 0.5 mg/ml Collagenase B (Invitrogen) in 37°C for 20 min rounds until the hearts were completely dissociated. Detached cells were re-suspended in PBS/20% FCS and kept at +4°C with gentle agitation until staining or FACS isolation.

### BioMark Real-Time PCR Fluidigm

#### Single cell analysis

A 96 well plate was prepared, by adding 5 μl master mix containing 2.5 μl CellDirect 2x Reaction mix, 0.6 μl RT/Taq mix (CellDirect One-Step qRT-PCR Kits (Invitrogen), 0.05 μl SUPERase-In RNase Inhibitor (Ambion), 0.6 μl TE-buffer (Invitrogen) and 1.25 μl 0.2x Assay mix per well. The 0.2x Assay mix was prepared in advance by adding 1.5 μl of 20x TaqMan Gene Expression Assay (Applied Biosystems) each and adding TE-buffer to a total volume of 150 μl. Hearts from nkx2.5-eGFP heterozygous embryos ED11.5 and ED9.5 and wild-type control embryos were dissociated as described. Single cells were isolated using a FACS Aria III (BD Biosciences). The pre-amplification and cDNA production were performed using the MJ Research PTC-200 Thermo Cycler (Bio-Rad) as follows: 50°C for 900 s and 95°C for 120 s followed by 95°C for 15 s, 60°C for 240 s, for 22 cycles. The samples were diluted 4x with TE-buffer before storage in -20°C. Quantitative Real-Time PCR reactions (qPCR) were performed using the high-throughput BioMark Real-Time PCR system (Fluidigm, South San Francisco, CA). The 20x TaqMan Gene Expression Assays (Applied Biosystems) were diluted with an equal volume of 2x Assay Loading Reagent (Fluidigm). Pre-diluted single cell cDNA (2.7 μl) was mixed with 3.3 μl TaqMan Universal PCR Master Mix (2x) (Applied Biosystems) and 20x GE Sample Loading Reagent (Fluidigm). The chip was loaded with 5 μl of each mix for each cell and assay. The qPCR was run using the following protocol: an initial thermal mix at 50°C for 120 s, 70°C for 1,800 s and 25°C for 600 s followed by UNG and Hot start at 50°C for 120 s and 95°C for 600 s. Finally, 40 PCR cycles were performed at 95°C for 15 s and 60°C for 60 s.

Assays were chosen based on their relevance to cardiac expression, including transcription factors, functional genes and other established lineage markers, as well as for a pool of potential surface markers. The primers were selected from TaqMan hydrolysis probes qualified for qPCR (Life Technologies, Carlsbad, California) and chosen for minimum genomic DNA quantification and recommendations from the company. As it was not possible to make biological repeats due to the single cell nature of this experiment, technical repeats were made by analyzing the same template twice for each gene. Numerous non-template controls (NTCs) were included with the purpose of detecting possible background amplification. Positive control and controls without enzyme were included for verification of the method. The specific hydrolysis probes used for cDNA amplification during the Fluidigm and qPCR experiments can be found in S1 Table.

#### Analysis of cell populations for extra-cardiac markers

Quantitative PCR was performed using the high-throughput BioMark Real-Time PCR system (Fluidigm, South San Francisco, CA) on a random selection of integrin population cDNA from wild type ED11.5 and ED9.5 and Nkx2.5-eGFP ED11.5 murine hearts. The template amount was kept as close to 50 cells/ μl as possible and the expression of the reference gene *Gapdh* was used to normalize for variations in RNA input. The protocol for the 96.96 gene expression assay was followed according to manufacturer’s specification. The specific hydrolysis probes used can be found in S1 Table.

### Immunofluorescence

Mouse embryos were fixed at 4°C with Stefanini solution; 20 g/L Paraformaldehyde, 6.667% saturated picric acid and equal amounts of Sörensen Buffer 0.2M, pH7.2 and distilled water, followed by equilibration in 20% sucrose prior to 10 μm cryo-sectioning. Sections were permeabilized in PBS/0.1% Triton X-100, blocked with 10% goat or donkey serum (Sigma-Aldrich) and stained with primary antibodies: chicken anti-mouse eGFP (Millipore), rabbit anti-mouse MYL2 (Synaptic Systems), rat anti-mouse ITGA6 Allophycocyanin (APC) (eBioscience), rat anti-mouse ITGA5 Biotin (LifeSpan Biosciences) and rabbit anti-mouse cTropT PE (BD Biosciences). Primary antibodies were visualized with secondary antibodies conjugated to *FITC/Cy3/Cy5* (Jackson ImmunoResearch) or *Alexa Fluor 555/647* (Invitrogen). Hoechst 33342 (Invitrogen) was used to localize nuclei. Imaging was performed using a Zeiss LSM 780 (Germany) laser scanning confocal microscope or a Leica DM500 B (Switzerland).

### Flow cytometry analysis and sorting

Embryonic nkx2.5-eGFP heterozygous and wild-type heart cell suspensions were stained in PBS containing 5% FCS with the following antibodies: rat anti-mouse Flk-1 Pacific Blue (BioLegend), rat anti-mouse CD49f (ITGA6) Allophycocyanin (APC) (eBioscience), hamster anti-mouse CD49a (ITGA1) PE (BD Biosciences), rat anti-mouse ITGA5, Biotin (LifeSpan Biosciences), sheep anti-mouse CDH2 (R&D) and/or rabbit anti-mouse cTropT PE (BD Biosciences). Streptavidin Qdot605 (Invitrogen) and donkey anti-sheep FITC (R&D) were used for secondary detection. To exclude non-viable cells, cells were stained with Fixable Viability Dye eFluor 780 (eBioscience). For intracellular staining, the cells were fixed in 4% PFA for 15 minutes, following by permeabilization in 0.1% Triton X-100 (3 minutes) and blocking in 1% BSA for 30 minutes. The cytometric acquisition and sorting was performed on Aria III (BD Biosciences) or Influx (BD Biosciences) using the 100 μM nozzle. Positive gates were set according to unstained, FMO and single stain controls. Small aliquots of cells were re-analyzed using the same settings for gating to assess purity.

### Quantitative Real-Time PCR reaction

CelluLyser buffer from Tataa Biocenter and the Roche Transcriptor First Strand cDNA Synthesis Kit were used for cDNA production. A maximum of 2,000 cells were sorted into 5.5 μl CelluLyser buffer with Protector RNase inhibitor and incubated at room temperature for 10 minutes. The cell lysate was frozen at -80°C o/n for increased lysis. The cDNA mastermix was comprised of: 0.1U/ μl dsDNAse, 2.5 μM Anchored-oligo (dT) Primer, 60 μM Random Hexamer Primer, 1X Transcriptor RT Reaction Buffer (5X), 1 mM Deoxynucleotide Mix, 0.5 U/μl Transcriptor Reverse Transcriptase and PCR-grade water, up to a total volume of 14.5 μl for each 5.5 μl cell lysate. cDNA synthesis was performed using the following program: 25°C for 600 s, 50°C for 1,800 s, 85°C for 300 s and cooled down to 4°C.

Quantitative PCR was performed using the Bio-Rad iQ5 Optical System and results were collected using the Bio-Rad iQ5 Software (Bio-Rad Laboratories) For the genes of interest, hydrolysis probes used can be found in S1 Table. The template amount was kept as close to 50 cells/ μl as possible and the expression of the reference genes *Ywhaz and Sdha* were used to normalize for variations in RNA input. The reaction master mix was prepared with either the TaqMan Gene Expression Master Mix (Invitrogen) or the iTaq Universal Probes Supermix (BioRad). On the Bio-Rad iQ5 Optical System, the qPCR TaqMan protocol was performed as follows; first 50°C for 120 s and 95°C for 600 s, followed by 40 PCR cycles with 95°C for 15 s and 60°C for 60 s. The qPCR BioRad protocol was performed as follows; first 95°C for 30 s, then, 40 PCR cycles with 95°C for 10 s and 60°C for 30 s. Absence of contamination was confirmed using non-template controls. In each gene-specific PCR, three biological replicates were compared in triplicate. The relative expression of each gene was calculated according to the 2^-ΔΔCT^ method using the GenEx (MultiD) software.

### Electrophysiology

Patch-clamp experiments were performed in the whole-cell configuration on spontaneously beating cardiomyocytes, as reported earlier [14]. Embryonic hearts ED11.5 were isolated and FACS-sorted as described, stored in DMEM +20% FCS + P/S + Glu and shipped to Germany. Cells were re-dissociated and functionally characterized 2 to 4 days after re-plating. Action potential (AP) recordings were obtained in the current clamp configuration, voltage ramps in the voltage-clamp mode by applying depolarizing pulses from -120 mV to 50 mV (length: 250ms). The pipette solution contained (in mM): 50 KCl; 80 Kasp; 10 EGTA; 10 Hepes; 3 MgATP; 1 MgCl (pH 7.4; adjusted with NaOH). The extracellular solution contained (in mM): 140 NaCl; 5.4 KCl; 1.8 CaCl2; 1 MgCl2; 10 Hepes; 10 Glucose (pH 7.4; adjusted with NaOH). The hormonal regulation was studied by applying acetylcholine (ACh, 10 μM) and Isoprenaline (ISO, 1 μM) via perfusion; the effect of agonists could be reversed by wash-out. The results were calculated in terms of percentage of variation of the agonist-induced chronotropic effect versus normal solution. Statistical analysis was performed using paired (agonist effects) or unpaired (AP characteristics) t-tests using Graph Pad Prism 5 (San Diego, USA) or LabChart 7 (AD Instruments, Spechbach, Germany) software. The results are expressed in mean ± s.e.m; a value of p < 0.05 was considered significant.

### Statistical analysis

Statistical analyses were performed using the SPSS software. Statistical analysis of mRNA expression between embryonic days and subgroups were compared with the Mann–Whitney U test and the chi squared test. A p < 0.05 was considered statistically significant. Tobit regression models were used for quantitative analysis of Fluidigm expression data. We note single-cell expression data can be viewed as a left-censored variable where expression values falling below the limit-of-detection for the device are unobserved, making the application of Tobit regression appropriate. A non-parametric bootstrapping approach was used to derive point estimates and 95% confidence intervals for differences in gene expression across groups; 10,000 bootstrap resamplings were performed to ensure stability of the estimates. Genes were considered to be differentially expressed between the two groups if the resulting 95% confidence interval for difference in log_2_ expression did not overlap zero. All regression analyses was performed with the R programming language [15] using the censReg package [16]. The qPCR data is depicted as mean relative expression ± s.e.m. Statistical significance was determined by the Kruskal-Wallis test with a paired Wilcoxon post-hoc analysis and with false discovery rate (FDR) adjustment. All further statistical analyses were performed with the GenEx (MultiD) software.

## Results

### Nkx2.5-eGFP and Cdh2 expression identifies developing cardiomyocytes in the mouse embryo

**Fig 1:**
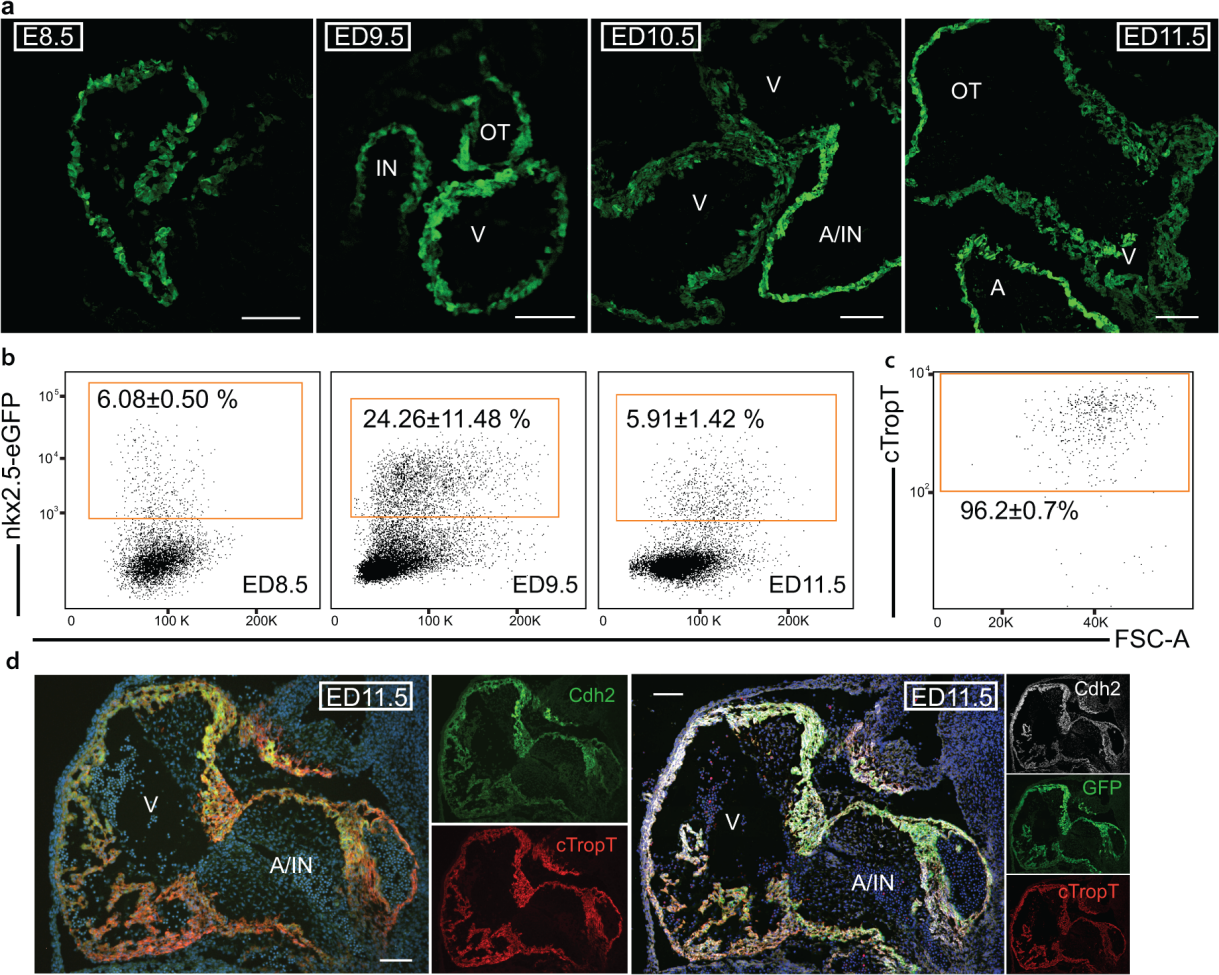
Nkx2.5-eGFP expression and CDH2 cardiac specificity is confirmed by flow cytometry and immunofluorescent staining. (a) Immunofluorescent visualization of Nkx2.5-eGFP on heart sections at embryonic day 8.5, 9.5, 10.5 and 11.5. (b) Percentage of eGFP positive (mean ± s.d) cells from mouse embryonic hearts at day 8.5 (n = 3), 9.5 (n = 6) and 11.5 (n = 14). (c) Percentage of cTropT positive cells from the Cdh2+ population of fixed ED11.5 (n = 4) wild type mouse hearts. (d) Immunofluorescent visualization of Cdh2 and cTropT on heart sections of wild type and Nkx2.5-eGFP hearts at embryonic day 11.5. Scale bar; 100 μm. Abbreviations: IN; Inflow, OT; Outflow, V; Ventricle and A; Atria.

Before the isolation of cells for single cell gene expression analysis, we confirmed the specificity of the markers that are used to isolate cardiomyocytes from developing embryonic hearts. We utilized the Nkx2.5-eGFP mouse [17], where eGFP expression is driven by a cardiac specific element of the Nkx2.5 promoter and will thus only be expressed in the embryonic heart before ED12 [17]. The expression of eGFP was examined by immunofluorescence at embryonic day (ED) 8.5, 9.5, 10.5 and 11.5. From the earliest heart tube, Nkx2.5-eGFP expression was detected throughout cardiac myocytes (Fig 1A). Nkx2.5-eGFP expression diminished after ED9.5, starting in the ventricles (Fig 1A), which is consistent with previous findings [17]. Flow analysis of isolated embryonic hearts confirmed that the eGFP^+^ percentage was highest at ED9.5 (24.26±11.48%, n = 6) compared to ED8.5 (6.08±0.50%, n = 3) or ED11.5 (5.91±1.42%, n = 14) (Fig 1B). In short, Nkx2.5-eGFP expression specifies cardiomyocytes at the earlier stages of mouse heart development. This was then used as a tool for selection of immature cardiomyocytes for further downstream analysis. Additionally, as we aimed to be less dependent on transgenic mouse models, we confirmed the cardiac expression of CDH2. Nkx2.5-eGFP and wild type ED11.5 hearts were analyzed for CDH2 and cTropT expression using both immunofluorescence and FACS analysis. Our results confirm previous findings [3] as we show that 96.2±0.7% (n = 4) of the wild type CDH2 positive cells express cTropT (Fig 1C and S4 Fig). Furthermore, 90.5±4.3% (n = 3) of the Nkx2.5-eGFP^+^ cells are positive for both CDH2 and cTropT when analyzed by flow cytometry (S4 Fig). We could also confirm expression of CDH2 in wild type and Nkx2.5-eGFP ED11.5 hearts by immunofluorescence (Fig 1D).

### Two distinct Myl2^+^ populations revealed by single cell profiling indicate a possible separation of ventricular and atrial myocytes

To investigate the heterogeneity of the embryonic heart, single eGFP^+^ cardiomyocytes were profiled using the 96.96 Dynamic Array chip. This method allows for simultaneous quantification of multiple genes in numerous single cells. We randomly sampled a total of 252 Nkx2.5-eGFP^+^ cells from mouse hearts ED9.5 and ED11.5 with an average sorting purity of 80±8.6% (n = 4). Each cell was screened for 48 genes (S1 Table and S1 Fig), including genes essential for cardiac function, ion channels, transcription factors, genes for other cell lineages, and more importantly, genes for membrane proteins. To be included in the analysis, detectable nkx2.5 expression was considered a prerequisite. At ED9.5 and ED11.5, 84 cells and 115 cells met this criterion, respectively.

**Fig 2:**
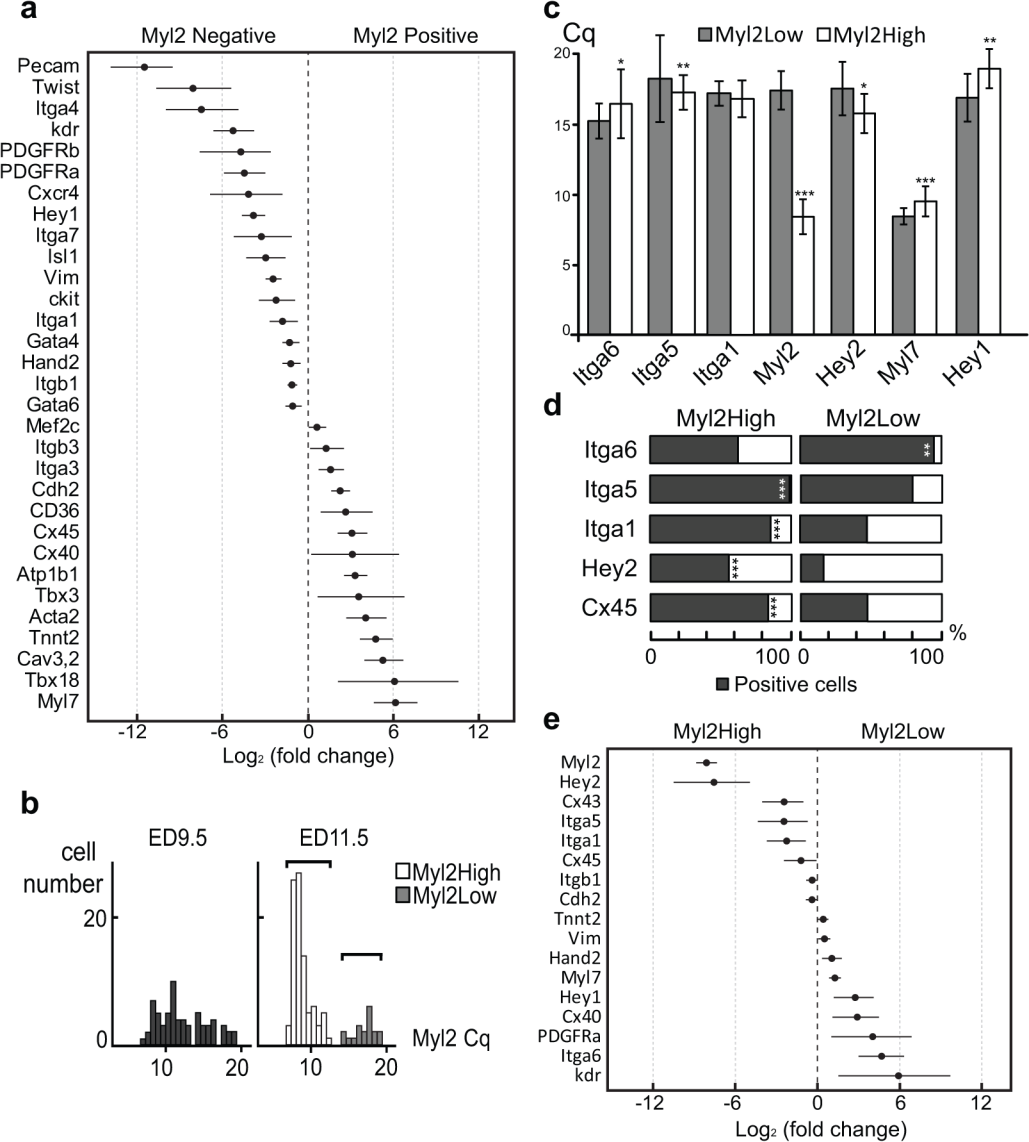
Single cell Fluidigm analysis revealed two *Myl2* positive populations with distinct expression profiles. (a) Selected log2 fold-change estimates between *Myl2* negative and positive cells at ED11.5 and ED9.5 with accompanying 95% confidence intervals. Comparisons were included in figure if 95% confidence interval did not overlap zero and at least 3 cells in each group expressed the gene of interest. (b) Rank values at ED9.5 and ED11.5 for *Myl2* expression revealed two different *Myl2* populations ED11.5 by Mann-Whitney U test. (c) Prominent differences in median Cq values at ED11.5 for the Myl2^High^ and Myl2^Low^ populations could be identified for *Itga6*, *Myl2*, *Hey2*, *Myl7* and *Hey1* by with the Mann–Whitney U test where *** P<0.001. (d) Proportion of positive cells ED11.5 between the Myl2High and Myl2Low group showed to be statistically significant for genes *Itga6*, *Itga5*, *Itga1*, *Hey2* and *Cx45*, *** P<0.001. (e) Selected log2 fold-change estimates between Myl2^Low^ and the Myl2^High^ group at ED11.5 with accompanying 95% confidence intervals. Comparisons were included in figure if 95% confidence interval did not overlap zero and at least 3 cells in each group expressed the gene of interest.

We then stratified cardiomyocytes based on Myl2 expression for subsequent gene expression analysis. Myl2 is a well-established marker for commitment to the ventricular cardiomyocyte lineage, and provides earlier discrimination than Myl7 [4, 5] (which, notably was expressed in all cells at ED11.5). Gene expression analysis of *Myl2* positive and negative cells at both ED9.5 and ED11.5 showed the *Myl2* ^+^ cells had a higher expression of *Myl7* (Log_2_ Fold-change = 6.17, 95% CI [4.7–7.6]), *cTropT* (Log_2_ Fold-change = 4.8, 95% CI [3.7–5.9]) and *Cdh2* (Log_2_ Fold-change = 2.3, 95% CI [1.7–2.9]) and as well other numerous genes critical for cardiomyocyte function, including *Atp1b1*, *Cav3*.*2*, *Tbx18*, *Cx40 and Cx45* (Fig 2A). The *Myl2* ^-^ population exhibited a number of gene expression shifts relevant to both lineage (cardiac vs. non-cardiac) and degree of differentiation. These included higher expression of Twist1 (Log_2_ Fold-change = 7.98, 95% CI [5.4–10.6]) [18], *Vim* (Log_2_ Fold-change = 2.4, 95% CI [1.9–2.9]), *Pecam* (Log_2_ Fold-change = 11.4, 95% CI [9.5–13.8]), *Pdgfra* (Log_2_ Fold-change = 4.4, 95% CI [2.7–7.5]), *Pdgfrb* (Log_2_ Fold-change = 4.4, 95% CI [3.0–5.8]), *ckit* (Log_2_ Fold-change = 2.2, 95% CI [0.9–3.4]) and *Flk1* (Log_2_ Fold-change = 5.2, 95% CI [3.8–6.6]) (Fig 2A).

Subsequently, we focused on the Myl2^+^ population at both time points. In analyzing Myl2^+^ cells, further investigation of a Mann-Whitney U revealed a bimodal distribution of Myl2 expression at ED11.5. A similar distribution could not be discerned at ED9.5 (Fig 2B), thus, indicating that the groups started to separate after ED9.5. These results directed our study to primarily focus on the later time point of ED11.5 where the presence of more mature cells and this separation of Myl2+ populations would be evident. Depending on their *Myl2* expression level at ED11.5, the two groups were named Myl2^High^ or Myl2^Low^. The expression level and number of positive cells for a range of cardiomyocyte and differentiation markers were compared between the two populations (Fig 2C and 2D). Myl2^High^ cells had a significantly higher expression level of Itga5, *Myl2* and the ventricular transcription factor *Hey2* while showing significantly lower expression of atrial *Myl7*, the atrial transcription factor *Hey1* and Itga6 (Fig 2C). Furthermore, the Myl2^High^ group had significantly more cells expressing *Itga1*, Itga5, *Hey2* and *Cx45* while significantly fewer cells expressed Itga6 (Fig 2D). Gene expression analysis revealed that the Myl2^High^ population had a higher expression of extracellular markers *Itga5* (Log_2_ Fold-change = 2.5, 95% CI [0.8–4.4]), *Itga1* (Log_2_ Fold-change = 2.25, 95% CI [0.9–3.7]), *Cx45* (Log_2_ Fold-change = 1.2, 95% CI [0.15–2.5]) and *Cx43* (Log_2_ Fold-change = 2.5, 95% CI [1.1–4.0]) as well as *Hey2* (Log_2_ Fold-change = 7.6, 95% CI [5.0–10.5]) and *Myl2* (Log_2_ Fold-change = 8.1, 95% CI [7.4–8.6]) (Fig 2E) while the Myl2^Low^ population had a higher expression of, amongst others, extracellular markers *Itga6* (Log_2_ Fold-change = 4.7, 95% CI [3.0–6.3]), *Cx40* (Log_2_ Fold-change = 2.9, 95% CI [1.1–4.4]) as well as *Myl7* (Log_2_ Fold-change = 1.3, 95% CI [0.9–1.7]) and *Hey1* (Log_2_ Fold-change = 2.7, 95% CI [1.2–4.1]) (Fig 2E).

Taken together, the cardiac chamber specific *Myl2*, *Hey2*, *Myl7* and *Hey1* profiles of the Myl2^High^ and Myl2^Low^ groups indicated a possible separation of ventricular from atrial myocytes. More interestingly, there was a clear association between integrin family members and Myl2 expression.

### Atrial cardiomyocytes are ITGA6^+^ITGA1^-^ITGA5^-^ at ED11.5

Differential expression analysis at ED11.5 showed Myl2^Low^ cells, most likely atrial-like cardiomyocytes, had higher expression of *Itga6* and lower levels of *Itga1* and *Itga5* relative to Myl2^High^.

Cells were isolated from Nkx2.5-eGFP^+^ ED11.5 murine hearts where qPCR analysis revealed that the presence of Itga6 selected for cells with a significantly higher expression of atrial *Myl7* and *Hey1* (S2 Fig). Conversely, low or absent expression of Itga6 was associated with a significantly higher expression of ventricular genes including *Myl2* and *Hey2* (S2 Fig).

**Fig 3:**
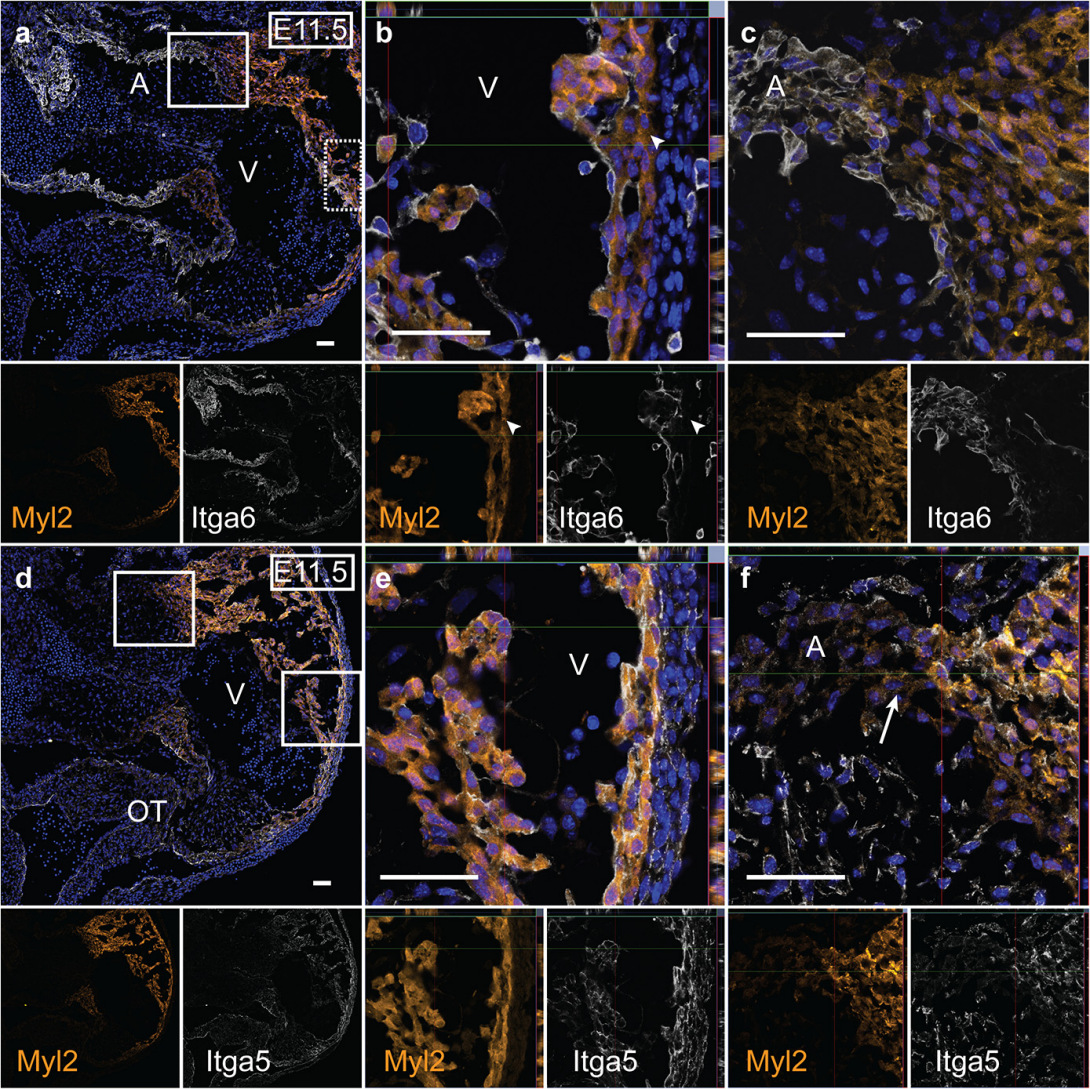
Localization of ITGA5 and ITGA6 in ED11.5 mouse hearts confirms proposed integrin expression patterns. (a-c) ITGA6 expression was found in atrial/inflow cells as well as in ventricular trabeculae, but could not be discerned in the compact ventricular myocardium (arrowhead). (d-f) ITGA5 expression was detected in ventricular cells with MYL2, as well as in the outflow tract, but not in the atrial regions (arrow). (n = 4) Green; Nkx2.5-eGFP, Orange; MYL2, White; ITGA5 or ITGA6. Scale bar; 50μm. Abbreviations: OT; Outflow, V; Ventricle and A; Atria.

**Fig 4:**
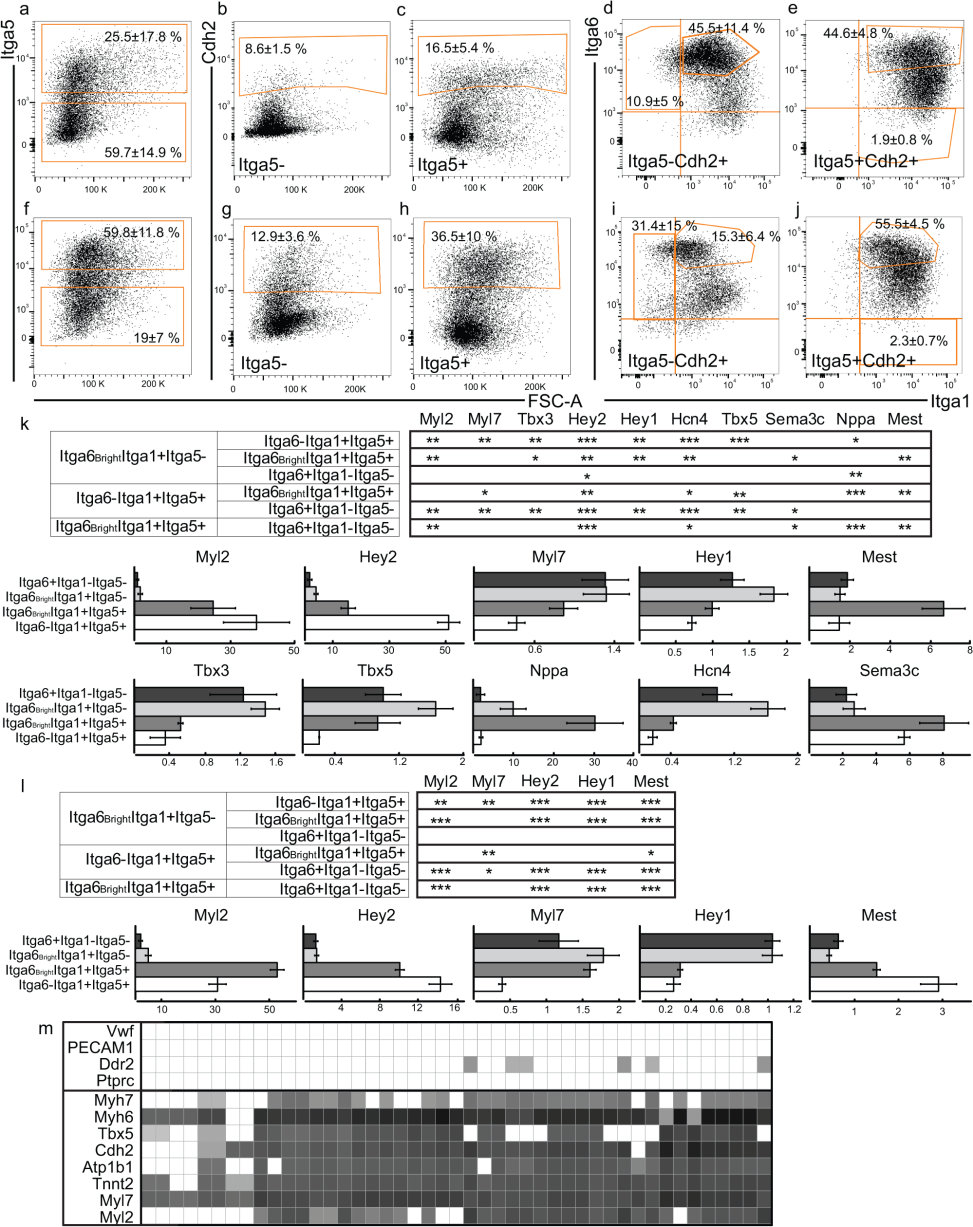
FACS and qPCR analysis reveals that integrin expression can separate atrial and ventricular populations. Flow cytometry plots of wild type mouse heart cells labelled with antibodies to CDH2, FLK1, ITGA5, ITGA6 and ITGA1 (a-e) ED11.5 (n = 8) and (f-j) ED9.5 (n = 4). (a and f) ITGA5 negative and positive cells were isolated and from these (b, g and c, h) CDH2 positive cells were selected for. (d/i) ITGA5^-^CDH2^+^ and (e/j) ITGA5^+^CDH2^+^ cells were interrogated based on ITGA6 and ITGA1 expression. Doublets, non-viable cells and FLK1 negative cells were gated away and the compensation and gates set based on single stains and FMOs. Populations ITGA6^+^ITGA1^-^ITGA5^-^, ITGA6^Bright^ITGA1^+^ITGA5^-^, ITGA6^bright^ITGA1^+^ITGA5^+^ and ITGA6^-^ITGA1^+^ITGA5^+^ isolated by FACS were analyzed for (k) *Myl2*, *Hey2*, *Myl7*, *Hey1*, *Mest*, *Nppa*, *Tbx3*, *Hcn4*, *Tbx5*, and *Sema3c* expression at ED11.5 (n = 3) and for (l) *Myl2*, *Hey2*, *Myl7*, *Hey1* and *Mest* expression at ED9.5 (n = 3). QPCR data depicted as mean relative expression ± s.e.m and p <0.05 was considered statistically significant. (m) Expression of multiple cardiac and non-cardiac genes as detected by Fluidigm analysis in a random selection of integrin population cDNA from wild type ED11.5 and ED9.5 and Nkx2.5-eGFP ED11.5 murine hearts. Delta Cq values for each well were computed relative to Gapdh, and wells negative for Gapdh were excluded from further analysis. For visual purposes, Delta Cq values for wells with no expression detected (Cq = 40) were set to the minimum Delta Cq value (white).

To confirm these findings on the protein level, ITGA5 and ITGA6 expression was explored in tissue sections (Fig 3 and S3 Fig). MYL2 and Nkx2.5-eGFP expression was used as ventricular and myocardial markers, respectively. At ED11.5, ITGA6 expression was observed in the inflow, primitive ventricular trabecular and all atrial cells (Fig 3A–3C). Conversely, ITGA5 was only expressed in the outflow, compact layer and trabecular ventricular cardiomyocytes ED11.5, leaving the inflow/atria negative (Fig 3D–3F). Additionally, these subpopulations of cells were isolated by FACS and qPCR was used to confirm cardiac identity of the sorted cells. Specifically, we isolated ITGA6^+^ITGA1^-^ITGA5^-^ and ITGA6^bright^ITGA1^+^ITGA5^-^ cells from wild type mice at ED9.5 and ED11.5 with an average purity of 94.9±2.2% (n = 5) (Fig 4A–4J and S4 Fig). These populations had a significantly higher expression of atrial genes *Myl7* and *Hey1* as well as of the conduction genes *Tbx3* and *Hcn4* at ED11.5. In addition, they demonstrated lower levels of ventricular markers *Myl2* and *Hey2* and outflow/pulmonary [19] myocardial marker Sema3c (Fig 4K). Similarly, at ED9.5, these cells had a significantly high expression of *Hey1* while expressing low levels of both *Myl2* and *Hey2* (Fig 4L). When comparing the ITGA6^+^ITGA1^-^ITGA5^-^ and ITGA6^bright^ITGA1^+^ITGA5^-^ populations at ED11.5, ITGA6^bright^ITGA1^+^ITGA5^-^ cells showed a significantly higher expression of *Hey2* and trabecular marker *Nppa* [20].

### Ventricular compact myocardium is ITGA6^-^ITGA1^+^ITGA5^+^ while trabecular cardiomyocytes are ITGA6^+^ITGA1^+^ITGA5^+^at ED11.5

Differential expression analysis at ED11.5 showed that the candidate ventricular Myl2^High^ cells exhibited higher expression of Itga1 and Itga5 and lower levels of Itga6 relative to Myl2^High^. Additionally, in vivo localization of Itga6 and Itga5 at ED11.5 revealed that the ventricular compact and trabecular myocardium layers displayed opposing expression patterns. ITGA5 expression was identified in both the compact and trabecular ventricular myocardium and outflow tract at ED11.5 (Fig 3D–3L). ITGA6 expression was, besides the atria, only observed in the trabecular ventricular myocardium, with a gradual decreased expression towards the compact layer, which was negative (Fig 3A–3C).

Thus, we isolated two populations; ITGA6^-^ITGA1^+^ITGA5^+^ and ITGA6^bright^ITGA1^+^ITGA5^+^ (Fig 4E and 4J) from wild type mice. Quantitative PCR analysis confirmed these two populations as having a ventricular profile by significantly higher expression of *Myl2* and *Hey2* and lower expression of *Hey1* at both ED9.5 and ED11.5 (Fig 4k and 4L). Moreover, the ITGA6^-^ITGA1^+^ITGA5^+^ cells showed significantly higher expression of ventricular *Hey2* and lower expression of *Hcn4* at ED11.5 and *Myl7* at both ED9.5 and ED11.5 (Fig 4K and 4L). Starting at ED11.5, both *Mest* and *Nppa* expression becomes restricted to trabecular myocytes[20, 21], confirming the trabecular origin of ITGA6^bright^ITGA1^+^ITGA5^+^ cells as these had a significantly higher expression of *Mest* and *Nppa* ED11.5 (Fig 4k) than ITGA6^-^ITGA1^+^ITGA5^+^ ED11.5 but not ED9.5 (Fig 4L).

### Integrin-defined cardiomyocytes are fully functional and exhibit lineage specific phenotypes

In addition to verifying Cdh2 as a good pan-cardiomyocyte marker, we confirmed that the expression of non- cardiac genes were below detection limit in all the isolated populations. A randomized selection of cDNA from wild type ED9.5, ED11.5 and Nkx2.5-eGFP ED11.5 murine integrin populations was screened for the expression of cardiac and non-cardiac genes (Fig 4M). No expression of endothelial cell markers *Vwf*, *Pecam1* or hematopoietic *Ptprc* (CD45) could be detected and only a few populations had any detectable expression of *Ddr2* (almost exclusively expressed in non-cardiomyocytes during embryonal development [22]). On the other hand, all populations showed expression of at least one of the cardiac genes *Myh6*, *Myh7*, *Atp1b1*, *Myl2*, *Myl7*, *Cdh2*, *Tbx5* and *cTropT* (Fig 4M).

**Fig 5:**
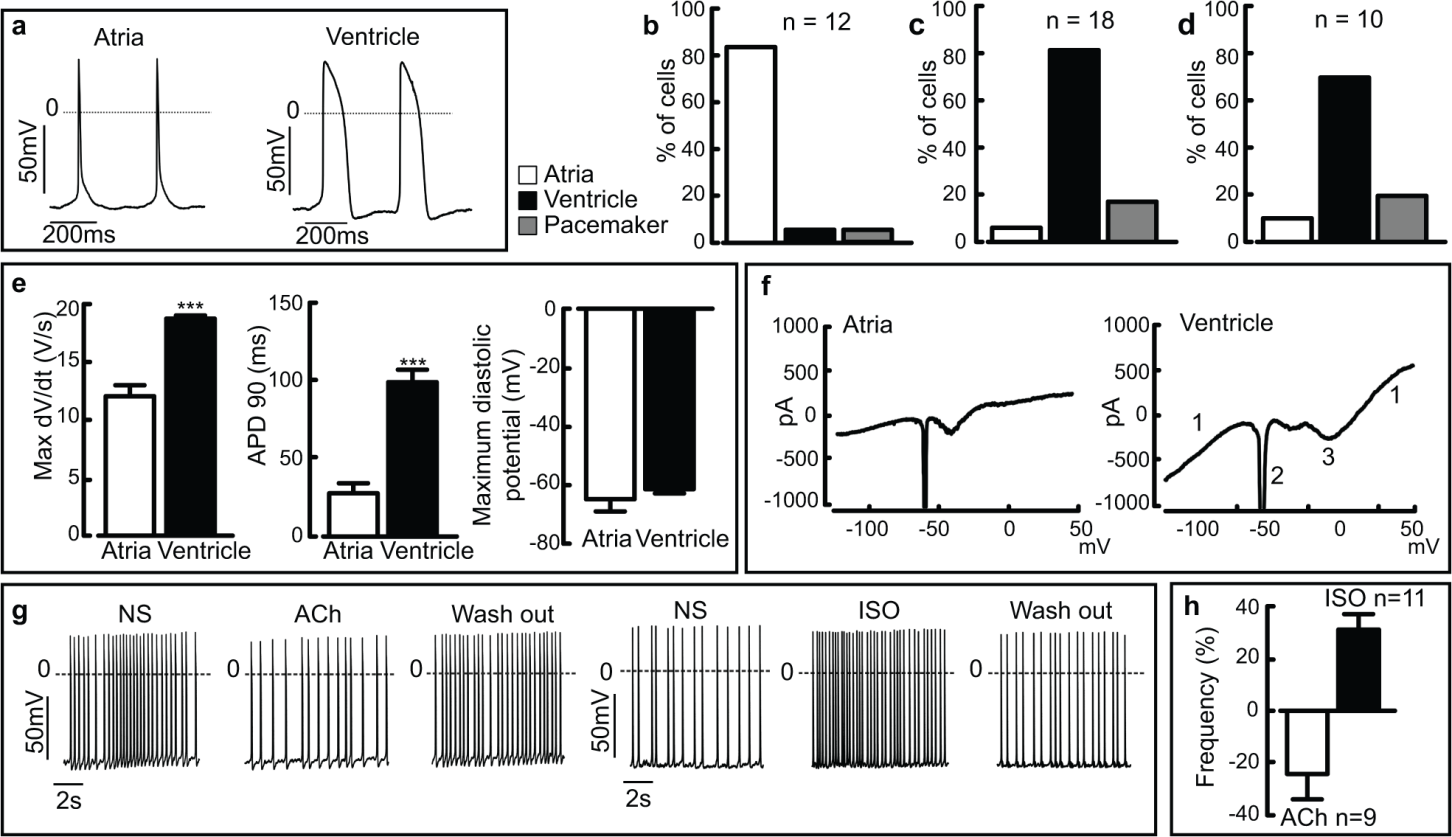
Subtype characterization and hormonal modulation of isolated cardiomyocytes support the phenotypes suggested by genetic profiling. (a) Representative action potentials (APs) of typical atrial and ventricular-like cells. (b) The ITGA6^+^ITGA1^-^ITGA5^-^ sorted population reveals a prominent enrichment of atrial-like cells (n = 12), whereas both the (c) ITGA6^-^ITGA1^+^ITGA5^+^ (n = 18) and (d) ITGA6^bright^ITGA1^+^ITGA5^+^ (n = 10) sorted populations reveal ventricular-like cardiomyocytes. (e) Analysis of key AP parameters shows clear differences between the atrial- and ventricular-like cardiomyocytes. (f) Representative voltage ramp recordings from an atrial and a ventricular-like cardiomyocyte. Note the functional expression of inward and outward current components, namely voltage dependent K^+^–(1), Na^+^–(2) and L-type Ca2^+^ (3)–currents. (g) Representative AP traces of ventricular-like cardiomyocytes and their response to perfusion with ACh (10 μM) (left traces) or Isoprenaline (1 μM). Note the negative and positive chronotropic response, respectively, and its reversal upon wash-out of the agonists. (h) Statistical analysis of the negative and positive chronotropic effects expressed as % of frequency variation in presence of the respective agonist compared to normal solution. Abbreviations: (APD90) action potential duration at 90% of repolarization; (Max dV/dt) Maximum rate of rise of the action potential; (NS) normal solution, (ISO) Isoprenaline, (ACh) Acetylcholine. The results are expressed as mean ± s.e.m. *** *P* <0.001 significant difference between the atrial and ventricular-like cells.

In order to prove cellular integrity and physiological function of the FACS-sorted cardiomyocytes and to characterize their cellular subtypes, patch clamp experiments were performed. A vast majority of the ITGA6^+^ITGA1^-^ITGA5^-^ cardiomyocytes (83.3%, n = 12) displayed action potential (AP) shape and short AP duration typical for atrial-like cells (Fig 5B), only two cells did not. In contrast, the ITGA6^-^ITGA1^+^ITGA5^+^ and ITGA6^bright^ITGA1^+^ITGA5^+^ populations revealed a prominent enrichment (82.30%, n = 18 cells and 70%, n = 10, respectively) for ventricular-like cells showing typical AP shape and longer AP duration (Fig 5C and 5D). Within each of these, only three cells did not display a typical ventricular shape. Interestingly, two cells within the ITGA6^bright^ITGA1^+^ITGA5^+^ population displayed pacemaker-like phenotypes (Fig 5D).

Key parameters of the ITGA6^+^ITGA1^-^ITGA5^-^ and ITGA6^-^ITGA1^+^ITGA5^+^ APs were analyzed (mean±s.e.m): maximum rate of rise of the AP (dV/dt; atrial cells: 11.9±0.9V/s; ventricular cells: 18.5±0.3V/s), AP duration at 90% of repolarization (APD90; atrial cells: 27.7±5.7ms; ventricular cells: 100.55±7.7ms) and maximum diastolic potential (MDP; atrial cells: -65.2±4.1mV; ventricular cells: -61.8±1.3mV) (Fig 5E). Thus, our data demonstrate clear differences between atrial-like and ventricular-like APs for maximal dV/dt (unpaired t test p>0.001) and for APD90 (unpaired t-test p>0.001) (Fig 5E). By applying voltage ramps we could detect expression of inwardly- and outwardly rectifying K^+^-currents as well as voltage dependent Na^+^- and Ca^2+^-currents in both the atrial and ventricular populations (Fig 5F). These results are in accordance with previous electrophysiological studies on murine embryonic cardiomyocytes [1, 23].

Key signaling pathways in the compact layer ventricular-like cells (ITGA6^-^ITGA1^+^ITGA5^+^) were investigated and their response to muscarinergic and adrenergic agonists assessed in the current-clamp mode. Application of acetylcholine (ACh, 10μM) resulted in a prominent negative chronotropic response with a decreased AP frequency by -24.2% (n = 9, paired t test p = 0.0235) (Fig 5G and 5H). Isoprenaline (ISO 1μM), a β-adrenergic agonist, strongly increased the AP frequency in all cells tested by 30.8% (n = 11, paired t test p = 0.0009) (Fig 5G and 5H). Both the muscarinergic and adrenergic effects could be reversed by wash-out (Fig 5G).

## Discussion

Due to the technical limitations associated with single cell isolation and analysis, current understanding of cardiomyocyte development has mainly been achieved by lineage tracing and transplantation strategies. We have previously shown that functional early embryonic cardiomyocytes can be FACS-isolated based on surface markers [1]. In this paper we move a step further and use single cell high throughput screening for the identification of extracellular markers for different cardiomyocyte cell populations during heart development. We show that Itga6, Itga1 and Itga5 can be used to isolate fully functional cells from the atria, the compact ventricular layer and the trabecular ventricular compartment with minimal non-cardiac cell contamination.

With the use of Nkx2.5 cardiac-enhancer driven eGFP we isolated early fetal mouse cardiomyocytes. This population is however, similarly to VCAM-1^+^ cells [1], still very heterogeneous and a traditional microarray would hide smaller populations. We therefore used single cell qPCR for a high resolution exploration of cellular differences. By linking functionally important intracellular genes to extracellular markers we strived to identify combinations that would enable live isolation and purification of cardiac sub-populations.

In Nkx2.5-eGFP^+^ murine cells we identified two *Myl2* ^+^ populations that had either a high or low expression profile at ED11.5, in contrast to ED9.5, thus indicating an important branching point in development. By associating the *Myl2* ^+^ populations to intracellular and surface marker expression, we observed a distribution and segregation of atrial and ventricular cell markers. This was accompanied by differential integrin expression that we utilized for the live isolation of the populations. Moreover, this capacity of integrin defined subtype separation could be confirmed in wild-type, non-transgenic mice by selecting for Cdh2.

Atrial cells maintained an ITGA6 expression throughout early cardiac development while not expressing ITGA1 or ITGA5. Later, atrial ITGA5 expression could be discerned at ED13.5 and by ED18 all of the atria were ITGA5^+^ (S3 Fig). From ED13.5 and onwards, ITGA6 expression in the atria was increasingly intensified in the atrial trabecular cells (S3 Fig). Thus, the surface marker pattern of ITGA6^+^ITGA1^-^ITGA5^-^ reflects the atrial population early in development. Furthermore, the atrial trabecular cells have to be taken into consideration. These cells were ITGA6^+^ITGA5^-^ at ED11.5 as seen by immunofluorescence (Fig 3). Cells that fell into this expression profile could either be ITGA1^+^ or ITGA1^-^. We have shown that the ITGA6^+^ITGA1^-^ITGA5^-^ population is of atrial origin. In comparison, the ITGA6^bright^ITGA1^+^ITGA5^-^ population showed a significantly higher expression of trabecular marker *Nppa* than the atrial population at ED11.5 (Fig 4K). Additionally, it had a significantly higher expression of conduction markers *Hcn4* and *Tbx3* compared to the all ventricular populations. This suggests that the expression of Itga1 within the atrial population could select for a mixed population containing conduction system cells. However, in order to verify this, further study of this population is needed.

Initially during heart development, the ventricles are dominated by trabecular myocardium. As the embryo grows, however, the compact layer will expand and become the major contractile force in the heart [24]. We were able to differentiate between ventricular compact and trabecular cells among ITGA1^+^ITGA5^+^ cells by means of the absence (compact) or presence (trabecular) of ITGA6, as was previously suggested by Collo *et al* [11]. At both ED9.5 and ED11.5, murine trabecular ventricle cells expressed ITGA5 and ITGA6 as seen by immunofluorescence (Fig 3A–3F and S3 Fig) well as ITGA1, as confirmed by FACS on ED11.5 cells (Fig 4A–4E and 4k). From ED13.5 and forward, ITGA6 expression gradually became restricted to the innermost trabecular cells and trabecular ITGA5 expression is seemingly lost at ED13.5, only to be regained by ED18 (S3 Fig). It has been suggested that integrin expression is related to cell cycle stage as the fibronectin receptor contains the ITGA5 subunit and its expression contributes to maintain a proliferative state in skeletal muscle. Consequently, increased ITGA5 expression at ED18 comes at a time when both atrial and ventricular cardiomyocytes expand prior to birth. ITGA6 expression, on the other hand is thought to support differentiation [12].

As indicated, the triple-positive cell population (Fig 4E and 4J) would encompass trabecular ventricle cells. This was confirmed as ED11.5 ITGA6^bright^ITGA1^+^ITGA5^+^ cells had a significantly higher expression of *Mest* (Fig 4k), a genes expressed in trabecular cardiomyocytes [20, 21] (Fig 4D and 4L). This could not be discerned at ED9.5 (Fig 4F–4J and 4L) as *Mest* becomes restricted to trabecular cells at a later point [20, 21]. It has previously been suggested that the conduction system has its origin in the trabecular cell population [4]. Indeed, the ITGA6^bright^ITGA1^+^ITGA5^+^ population had a significantly higher expression of Nppa, a gene associated with the ventricular conduction system, as well as higher expression of *Hcn4* (Fig 4K), supporting that the conduction system could be developmentally related to the trabecular myocardium. While the electrophysiological measurements on ITGA6^bright^ITGA1^+^ITGA5^+^ cells showed a predominantly ventricular profile (Fig 5D), it also showed a tendency towards a higher proportion of pacemaker-like cells. This suggests that further in-depth study of this population is required, as it is highly likely that this population is comprised of multiple cell types.

The ventricular compact layer maintains ITGA6^-^ITGA5^+^ identity throughout fetal development (Fig 3 and S3 Fig). The ITGA6^-^ITGA1^+^ITGA5^+^ cells expressed significantly higher levels of *Myl2* and *Hey2* at both time points (Fig 4K and 4L) and did not expand significantly in size from ED9.5 to ED11.5 (Fig 4E and 4J), which is consistent with the compact ventricular cardiomyocyte origin. The possibility of compact layer cell isolation is an important milestone in cardiac surface marker identification. As it is well accepted that compact layer cardiomyocytes have a higher proliferation capacity compared to trabecular cells during development [24], this could be of great interest for transplantation studies in infarction models.

This is the first report of murine atrial and ventricular cardiomyocyte population separation by the use of integrin expression. We show that the compact layer can be separated out from the remaining ventricular myocardium. Furthermore, as atrial cells are virtually absent from this population, the risk of arrhythmias upon transplantation could be greatly reduced.

The discovery of lineage specific integrin patterns could provide scientists with an easy and reliable method for the isolation of pure populations usable for stem cell based therapies in heart disease. These markers will be of great value for future developmental studies of cardiomyocytes and the heart as a whole.

## Supporting Information

S1 Dataset Raw Cq values for the BioMark Real-Time PCR Fluidigm single cell analysis. (XLSX)

S1 Fig Fluidigm analysis of Nkx2.5-eGFP positive cells from ED9.5 and ED11.5 mouse hearts. (a) Boxplot per-sample of raw Cq values plotted to examine sample distribution and exclude time point bias (b) Raw per-gene distribution of Cq values broken across time points showed no abnormal Cq value distribution. Only expression values above the machine’s limit of detection are shown.
(EPS)

S2 Fig FACS and qPCR analysis results for Nkx2.5-eGFP ED11.5 mouse hearts. (a-j) Flow cytometry plots of hearts cells labelled with antibodies to FLK1, ITGA5, ITGA6 and ITGA1. (b-d) Doublets, non-viable cells and FLK1 negative cells were excluded. (e) Purity was accessed by re-analysis of investigated populations. (f) The ITGA5 negative and positive populations were isolated and (g and h) Nkx2.5-eGFP positive cells were isolated from each. (i) ITGA5-Nkx2.5-eGFP+ and (j) ITGA5+Nkx2.5-eGFP+ cells were interrogated based on ITGA6 and ITGA1 expression. All gates were based on single stains and FMOs. Populations ITGA6_bright_ITGA1^+^ITGA5^-^ (n = 3), ITGA6^-^ITGA1^+^ITGA5^-^ (n = 3), ITGA6_bright_ITGA1^+^ITGA5^+^ (n = 3), ITGA6_Dim_ITGA1+ITGA5+ (n = 2) and ITGA6^-^ITGA1^+^ITGA5^+^ (n = 2) isolated by FACS were analyzed for (k) Myl2, Hey2, Myl7, Hey1, Tbx3 and Hcn4. QPCR data depicted as mean relative expression ± s.e.m and p <0.05 was considered statistically significant for populations analyzed in triplicate (boxed).
(EPS)

S3 Fig Localization of ITGA5 and ITGA6 in the mouse heart ED9.5, ED13.5 and ED18. (a-f) ITGA6 expression can be localized to all atrial cells as well as the ventricular trabecular area at all time points. ITGA5 expression was localized to the inflow area (g-h) ED9.5 and to the compact ventricular cells together with MYL2 (i-j) ED13.5. (k-l) At ED18, ITGA5 is localized to the entire ventricles and most of the atria. Green; Nkx2.5-eGFP, Orange; MYL2, White; ITGA5 or ITGA6. Scale bar; 50μm
(TIF)

S4 Fig Fluidigm and population based cell isolation FACS plots. Representative isolation, analysis and purity analysis of cells used for the single cells Fluidigm (a-e) ED9.5 and (f-j) ED11.5 Nkx2.5-eGFP mouse hearts. (k-n) ED11.5 wild type analysis of fixed cells for Cdh2 and cTropT expression. (o-r) ED11.5 Nkx2.5-eGFP analysis of fixed cells for Cdh2 and cTropT expression.FACS isolation for qPCR analysis of (s-w) ED11.5 wild type cardiac cells with FACS purity analysis (x-aa) ED9.5 wild type mouse hearts.
(EPS)

S1 Table TaqMan hydrolysis probes used during the single cell Fluidigm experiments and for qPCR. (DOCX)
