# Supplementary figures and images for: Integrin Based Isolation Enables Purification of Murine Lineage Committed Cardiomyocytes
Source: PLoS One. 2015 Aug 31;10(8):e0135880. doi: 10.1371/journal.pone.0135880 (PMC4556377; doi:10.1371/journal.pone.0135880)

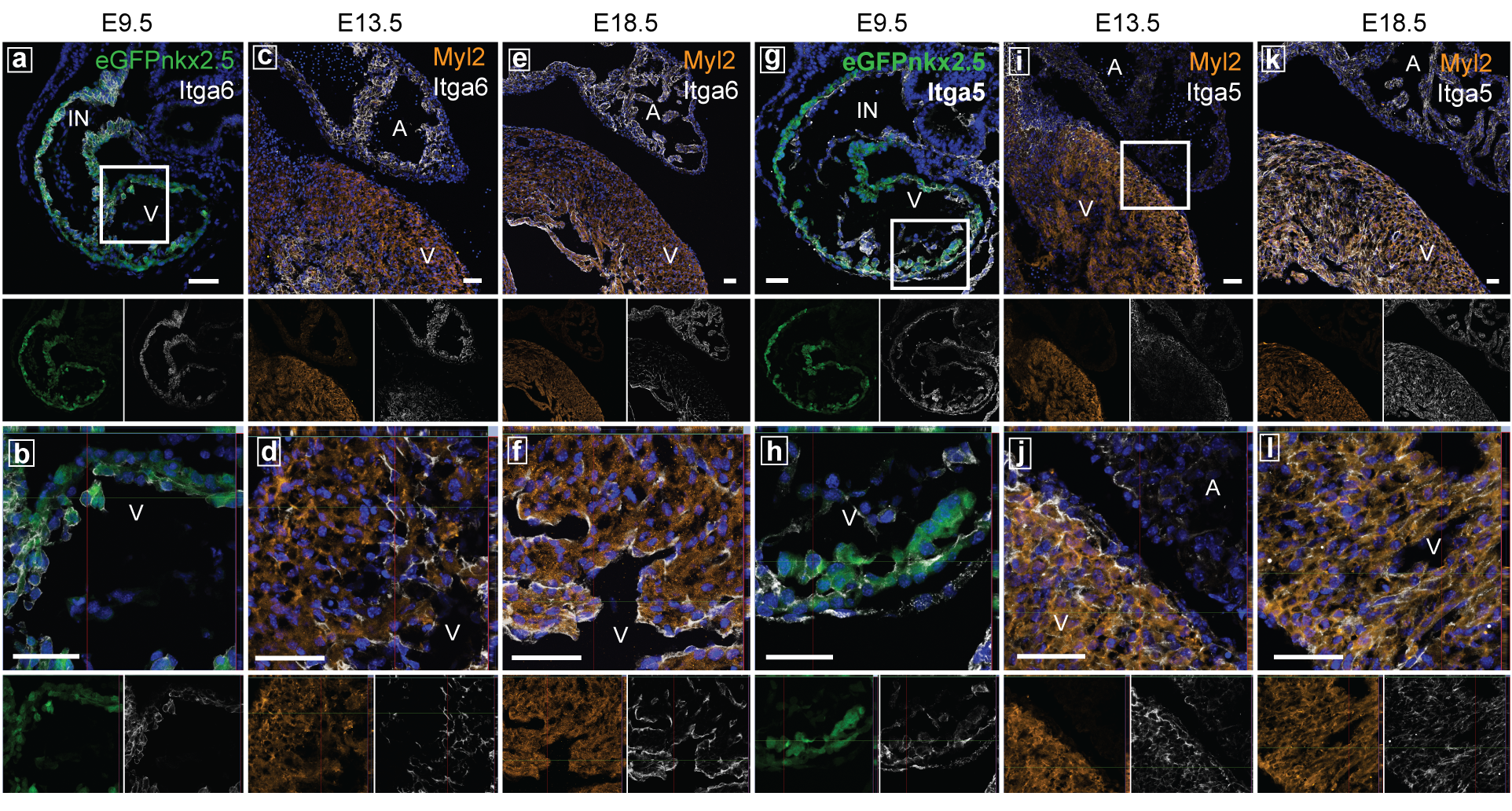

Supplement: S3 Fig — (a-f) ITGA6 expression can be localized to all atrial cells as well as the ventricular trabecular area at all time points. ITGA5 expression was localized to the inflow area (g-h) ED9.5 and to the compact ventricular cells together with MYL2 (i-j) ED13.5. (k-l) At ED18, ITGA5 is localized to the entire ventricles and most of the atria. Green; Nkx2.5-eGFP, Orange; MYL2, White; ITGA5 or ITGA6. Scale bar; 50μm (TIF) [file pone.0135880.s004.tif]
