# Supplementary material for: Integrin Based Isolation Enables Purification of Murine Lineage Committed Cardiomyocytes
Source: PLoS One. 2015 Aug 31;10(8):e0135880. doi: 10.1371/journal.pone.0135880 (PMC4556377; doi:10.1371/journal.pone.0135880)
Supplement: S1 Table — (DOCX) [file pone.0135880.s006.docx]

| **Gene** | **Assay ID#** |  | **Gene** | **Assay ID#** |
| --- | --- | --- | --- | --- |
| Acta2 | Mm01546133_m1 |  | Nppa | Mm01255747_g1 |
| Atp1b1 | Mm00437612_m1 |  | Pdgfra | Mm00440701_m1 |
| CD36 | Mm01135198_m1 |  | Pdgfrb | Mm00435546_m1 |
| Cntn2 | Mm00516138_m1 |  | Pecam1 | Mm01242584_m1 |
| Cdh2 | Mm00483213_m1 |  | CD45 | Mm01293575_m1 |
| Cx30.2 | Mm00731344_s1 |  | Sdha | Mm01352366_m1 |
| Cx43 | Mm00439105_m1 |  | Sema3c | Mm00443121_m1 |
| Cx45 | Mm01253027_m1 |  | Tbx3 | Mm01195726_m1 |
| Cx40 | Mm01265686_m1 |  | Tbx5 | Mm00803518_m1 |
| Cav3.2 | Mm00445382_m1 |  | Tbx18 | Mm00470177_m1 |
| Cxcr4 | Mm01292123_m1 |  | cTropT | Mm00441922_m1 |
| Ddr2 | Mm00445615_m1 |  | Twist1 | Mm00442036_m1 |
| Gata4 | Mm00484689_m1 |  | Vim | Mm01333430_m1 |
| Gata6 | Mm00802636_m1 |  | Vwf | Mm00550376_m1 |
| Hcn4 | Mm01176086_m1 |  | Ywhaz | Mm01158416_g1 |
| Hand2 | Mm00439247_m1 |  |  |  |
| Hey1 | Mm00468865_m1 |  |  | |
| Hey2 | Mm00469280_m1 |  |  |  |
| Isl1 | Mm00517585_m1 |  |  |  |
| Itga1 | Mm01306375_m1 |  |  |  |
| Itga2 | Mm00434371_m1 |  |  |  |
| Itga3 | Mm00442910_m1 |  |  |  |
| Itga4 | Mm00439770_m1 |  |  |  |
| Itga5 | Mm00439797_m1 |  |  |  |
| Itga6 | Mm00434375_m1 |  |  |  |
| Itga7 | Mm00434400_m1 |  |  |  |
| Itgb1 | Mm01253230_m1 |  |  |  |
| Itgb2 | Mm00434513_m1 |  |  |  |
| Itgb3 | Mm00443980_m1 |  |  |  |
| Flk-1 | Mm01222421_m1 |  |  |  |
| Kit | Mm00445212_m1 |  |  |  |
| Kcne2 | Mm00506492_m1 |  |  |  |
| Mef2c | Mm01340842_m1 |  |  |  |
| Mesp1 | Mm00801883_m1 |  |  |  |
| Mest | Mm00485003_m1 |  |  |  |
| Myh6 | Mm00440354_m1 |  |  |  |
| Myh7 | Mm00600555_m1 |  |  |  |
| Myl2 | Mm00440384_m1 |  |  |  |
| Myl7 | Mm00491655_m1 |  |  |  |
| Nkx2.5 | Mm01309813_s1 |  |  |  |
|  |  |  |  |  |
